# Low-Cost Cranioplasty—A Systematic Review of 3D Printing in Medicine

**DOI:** 10.3390/ma15144731

**Published:** 2022-07-06

**Authors:** Wojciech Czyżewski, Jakub Jachimczyk, Zofia Hoffman, Michał Szymoniuk, Jakub Litak, Marcin Maciejewski, Krzysztof Kura, Radosław Rola, Kamil Torres

**Affiliations:** 1 Department of Didactics and Medical Simulation, Medical University of Lublin, 20-093 Lublin, Poland; wojciech.w.czyzewski@gmail.com (W.C.); kamiltorres@wp.pl (K.T.); 2 Department of Neurosurgery and Pediatric Neurosurgery in Lublin, 20-090 Lublin, Poland; jakub.litak@gmail.com (J.L.); krzysztof_kura@wp.pl (K.K.); rola.radoslaw@gmail.com (R.R.); 3 Student Scientific Society, Medical University of Lublin, 20-059 Lublin, Poland; jakub.jachimczyk@protonmail.ch; 4 Student Scientific Association of Neurosurgery, Department of Neurosurgery and Pediatric Neurosurgery, Medical University of Lublin, 20-090 Lublin, Poland; michmatsz@gmail.com; 5 Department of Clinical Immunology, Medical University of Lublin, 20-093 Lublin, Poland; 6 Department of Electronics and Information Technology, Faculty of Electrical Engineering and Computer Science, Lublin University of Technology, 20-618 Lublin, Poland; m.maciejewski@pollub.pl

**Keywords:** cranioplasty, 3D cranioplasty, additive manufacturing, PMMA, PEEK, PLA, ABS, PET-G

## Abstract

The high cost of biofabricated titanium mesh plates can make them out of reach for hospitals in low-income countries. To increase the availability of cranioplasty, the authors of this work investigated the production of polymer-based endoprostheses. Recently, cheap, popular desktop 3D printers have generated sufficient opportunities to provide patients with on-demand and on-site help. This study also examines the technologies of 3D printing, including SLM, SLS, FFF, DLP, and SLA. The authors focused their interest on the materials in fabrication, which include PLA, ABS, PET-G, PEEK, and PMMA. Three-dimensional printed prostheses are modeled using widely available CAD software with the help of patient-specific DICOM files. Even though the topic is insufficiently researched, it can be perceived as a relatively safe procedure with a minimal complication rate. There have also been some initial studies on the costs and legal regulations. Early case studies provide information on dozens of patients living with self-made prostheses and who are experiencing significant improvements in their quality of life. Budget 3D-printed endoprostheses are reliable and are reported to be significantly cheaper than the popular counterparts manufactured from polypropylene polyester.

## 1. Introduction

**Chart 1:**
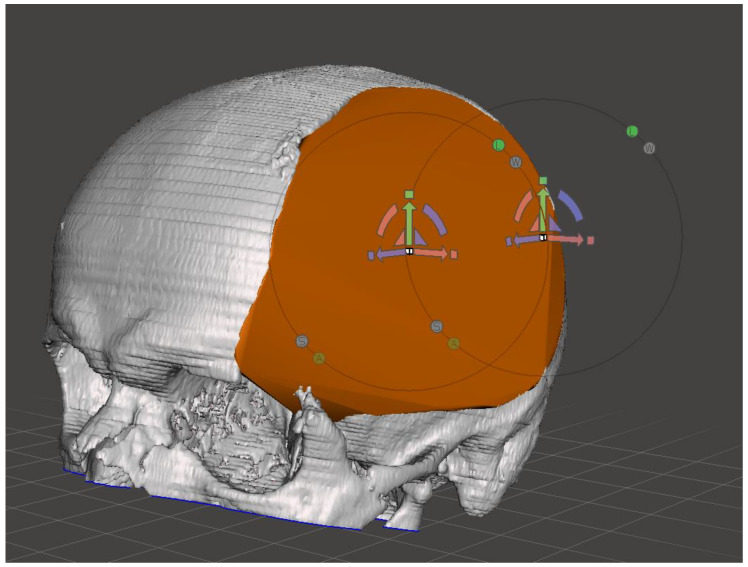
Cranial defect recreated using Autodesk^®^ Meshmixer.

The human skull is one of the most complex regions of the human body in terms of maintaining visually pleasing results. In the case of a post-traumatic or postoperative defect, cranioplasty requires constant medical development to provide a decent quality of life to affected patients by producing personalized endoprostheses [1]. Although widely adapted titanium endoprostheses often provide satisfactory results, they tend to be too expensive for low-income patients. Despite high biocompatibility, titanium leads to a significant artifact in CT and MRI imaging. Prostheses made of polypropylene–polyester are often used in cranioplasty, for which prices in the case of standard products (regular, oval shape) do not exceed USD 500 [2]. However, if there is a necessity to use a personalized prosthesis, the cost escalates to nearly USD 2000. In recent years, FDM- (fused deposition modeling) and SLA- (stereolithography) 3D-printing have become increasingly popular [3]. These methods provide a terrific opportunity for medicine to bring widely available solutions with inexpensive planning, prototyping, guiding, and even creating on-demand 3D-printed tools, models, and prostheses [4,5,6,7]. The personalized anatomical models can then be implemented in various engineering applications, such as the Materialise Mimics or Materialise 3-matic and CAD/CAM/CAE software, allowing their further analysis using, e.g., the finite element method (FEM) [8,9]. The materials used during these processes can be chosen accordingly, e.g., PET-G for surgical guides, PLA/resin for patient skull models, or PEEK for the production of prostheses. Implants are modeled in the CAD software (such as MeshLab^®^) using the STL files harvested from the DICOM data (Chart 1) (for example, by using RadiAnt^®^ or OsiriX^®^). This systematic review focuses on various utilizations of 3D-printing technology during surgical procedures in the craniofacial area and consists of a comparison of the multidisciplinary approaches that involve the manufacturing of surgical guides, skull models, partial cranial prostheses, CAD modeling, material analyses and case studies.

## 2. Methods of Article Selection

The search for articles, conducted on 15 April 2022, supporting the topic of our review, focused on the 3D-printing techniques employed in cranioplasty.

During the search, the following portals were used: PubMed, Scopus, Web of Science and Google Scholar. The searched articles were in English and not older than 7 years. The keywords used were “3D-printing cranioplasty”; “3D-printing endoprosthesis”; “3D-printing methods”; “3D-printing materials”; “3D-printing costs”; “3D-printing regulations”. These searched keywords resulted in 37,872 articles found. The screening inclusion and exclusion criteria provided at least 250 full-text articles suitable for this review. Inclusion criteria were at least one of: 3D printing in cranioplasty; 3D-printing costs in medicine; 3D printing of endoprostheses; comparison of 3D-printing methods; 3D-printing legal regulations. 

**Figure 1:**
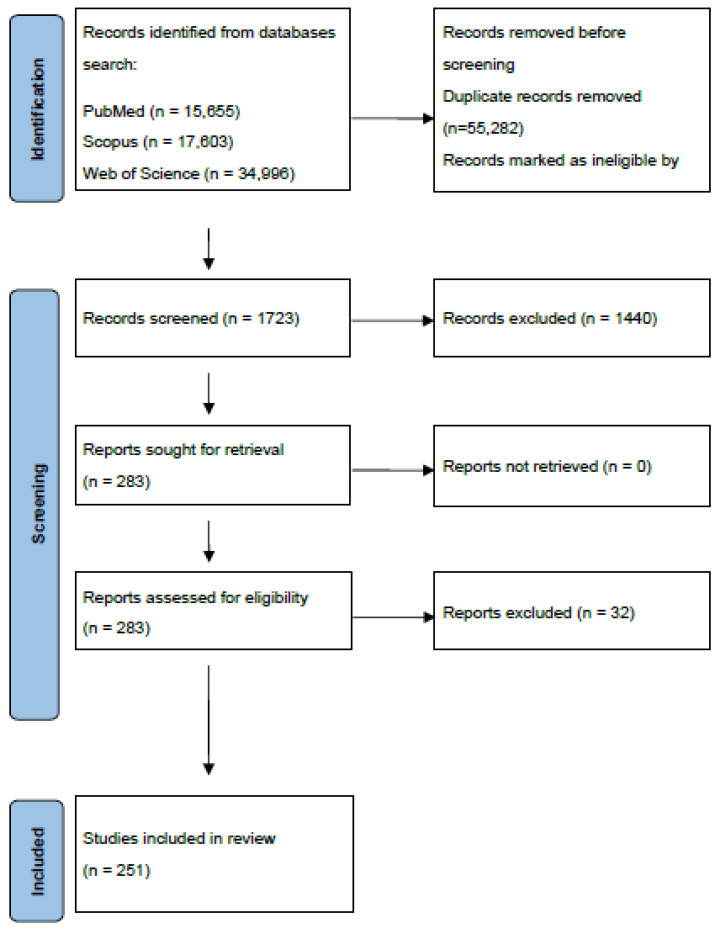
Flowchart of the identification and selection of studies according to the 2020 PRISMA statement.

Exclusion criteria: 3D printing of endoprostheses, which is much more expensive than the traditional methods; modeling of endoprostheses without the use of 3D printing; articles in a language other than English; duplicates; incomplete articles; bone endoprostheses other than those of the skull; 3D bioprinting with live cells; articles older than 7 years (Figure 1).

## 3. Methods of 3D Printing

Additive manufacturing employs the data computer-aided design (CAD) software to create 3D objects through the use of layer-by-layer various material depositions [10]. Furthermore, 3D printing encompasses several production technologies that involve solid (FDM—fused deposition modeling) [11], powder (SLS—selective laser sintering, SLM—selective laser melting) [12,13,14] or liquid-based materials (SLA—stereolithography, DLP—digital light processing) [15,16,17]. They differ in the manner that they arrange elements, as well as in their surface finish, operating time, material selection, durability, and value (Table 1). 

### 3.1. Fused Deposition Modeling (FDM)

**Chart 2:**
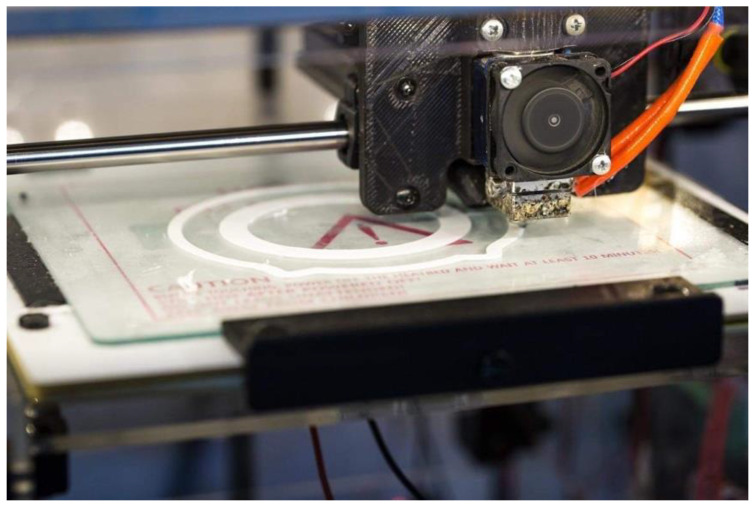
FDM 3D Printer.

FDM was invented in 1988 by Scott Crump—a co-founder of Stratasys^®^ [39]. This method is extrusion-based, where thermoplastic polymers in the form of filaments are wound on spools and are fed to the printer’s nozzle, where, after heating and melting, are subsequently selectively distributed layer-by-layer in accordance with the outline of the geometric model previously processed by the 3D-printing software (Chart 2) [40,41]. The characteristic of printing depends on the material’s dropping pressure, size of the nozzle, as well as the rate of feeding [42,43].

FDM has become increasingly prevalent of late, due to its immense accuracy, simplicity, relatively low price and eligibility for house printing [44,45]. It also utilizes strong engineering-grade materials that include PLA, ABS, PET-G and PP, among others [46]. Further materials worth mentioning in the implants market comprise PMMA, PEEK, or PVDF [19]. It is worth mentioning here, that the fused filament fabrication of metal is capable of fabricating patient-specific implants made out of titanium alloy [47].

FDM-based additional manufacturing is widespread in medicine and frequently utilized in the production of models that aid in clinical diagnoses and preoperative planning [20,21,22,48]. FDM enables the design and production of bone scaffolds and implants, and takes part in drug development research [24,25,26,27,28,49]. Novel literature also presents numerous examples where it was used to prepare cranioplasty implants [29,30,31,32]. For example, Thakur et al. used an FDM printer to prepare an ABS-made template for subsequent PMMA-implant fabrication, reporting high accuracy, precision, and excellent esthetics, which were obtained postoperatively [50]. However, in this indication, PCL and PLA belong to most manufactured polyesters used in FDM printers [51]. 

### 3.2. Selective Laser Melting (SLM)

**Chart 3:**
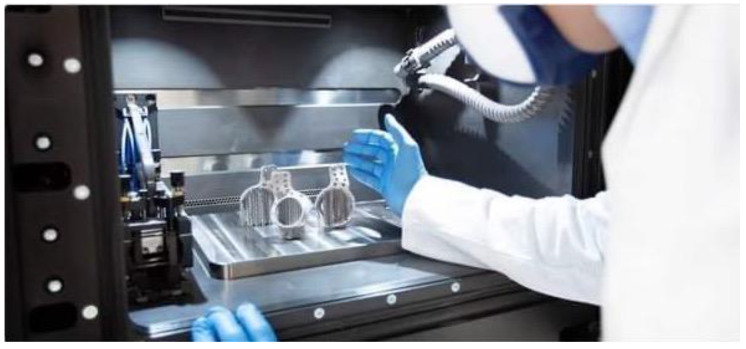
SLM 3D Printer (Source: Sandvik).

SLM is a powder-bed fusion technology that uses a powdered metallic alloy that is processed with a high-density-focused laser beam, layer-by-layer [52], building a compact three-dimensional structure characterized by considerable hardness and low porosity [53]. The novel literature presents numerous examples of research dedicated to the type of materials constructed by SLM (Chart 3). Among them, the most employed are titanium-, nickel-, and ferrous-based alloys [54]. The SLM-manufactured titanium 3D-prostheses perfectly emulate the biomechanical properties of natural human bone due to its precise internal and external architecture [55]. It is characterized by a high resistance to rusting [56] and its mechanical strength at room temperature [57]. This method is often used in modern medicine due to the high durability of Ti, radiolucency, and the minimal risk of post-treatment infections compared to other artificial materials [58]. Additionally, 3D-printed Ti prostheses are already utilized in the production of hip prostheses [18], but are also applicable in 3D-cranial reconstruction [46,59]. 

The SLM-titanium-made prostheses represent an interesting direction for the cranial reconstruction market; however, their high manufacturing costs discourage its widespread use by patients and hospitals of a lower socioeconomic status [60]. 

### 3.3. Stereolithography (SLA)

SLA is a solid free-form (SFF) technique that uses a photosensitive polymer resin that reacts under the influence of a computer-controlled laser beam. The illumination-induced chemical reaction causes the formation of polymers by a cross-link fusion of chemical particles (monomers and oligomers) [49]. A previously designed three-dimensional project is converted into a stack of two-dimensional images. Then, a computer-derived pattern is illuminated on the surface of a liquid resin. Each 2D layer is then photochemically solidified, layer-by-layer with the use of a building stage and moving laser beam to form a desired three-dimensional solid representation of the pre-programmed CAD-CAM model. Subsequent solvent cleansing of wet resin from the model surface is then performed [61,62]. 

Stereolithography presents an advantage over other printing methods through its greater speed, controlled integrity, high-print resolution, and heat-free printing [63,64]. 

**Chart 4:**
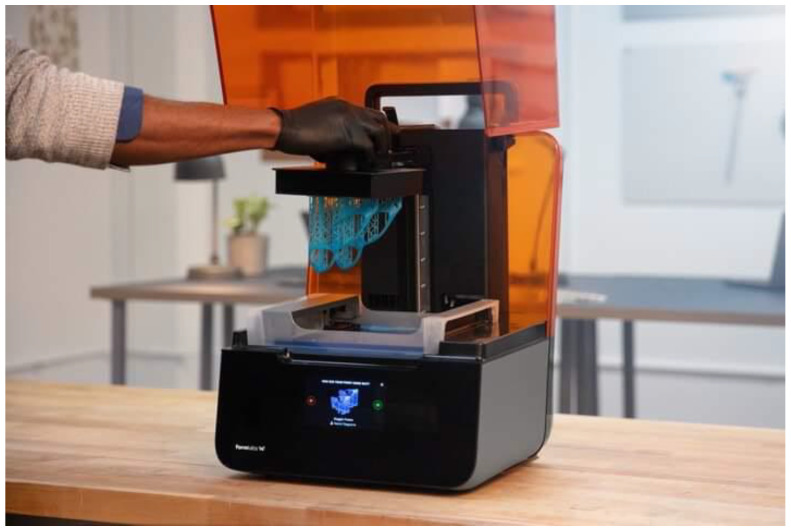
SLA 3D Printer (Source: Formlabs).

The method was patented by Charles W. Hull in 1986 [28], and in addition to industrial applications, stereolithography is now widely used in various fields of medicine (Chart 4). Polyethylene glycol (PEG), poly (D, L-lactide) (PDLLA), poly-ε-caprolactone (PCL), and poly(propylene fumarate) (PPF) comprise the most common photopolymerizable resins for tissue-engineered implants [65]. SLA finds its application in the medical field to create personalized endocardial implants [33], accurate models of reconstructing intestinal epithelial architecture [34], and innovative treatment of focal cartilage lesions [35,66]. SLA-3D printing has also been used to improve a cranioplasty procedure’s performance. Pöppe et al. described the technique of creating a patient-specific SLA-printed template for subsequent PMMA-implant modeling. In 14 surgeries, all cranioplasties perfectly matched the patient’s anatomy. Out of those, the three patients who bore clinically relevant coagulation-related risk factors required revision surgery due to a postoperative hematoma. Apart from that, no other complications have been recorded [67]. The analogous method was used by da Silva Junior, where a polycarbonate-made SLA-printed two-piece mold accompanied an intraoperative PMMA final implant formation, achieving satisfying results without any complications on a 3–26-month follow-up [68]. A similar method has also been reported by other authors [51].

### 3.4. Digital Light Processing (DLP)

DLP is a technique analogous to SLA, with the difference being that instead of using a moving laser beam, the entire layer is cured simultaneously by a onetime projection of the whole image in an x/y space into photosensitive liquid resin, which guarantees excellent dimensional accuracy by avoiding the quality losses caused by a traveling laser [69,70]. The mechanical strength of the objects manufactured with the use of resin in this technology is sufficient to produce exoprostheses used in the rehabilitation of hands and wrists [36]. It can also be applied in the production of dentures [37]. 

### 3.5. Selective Laser Sintering (SLS)

SLS is a 3D-printing technology that involves a powder bed for an object’s construction analogously to SLM. However, in contrast to SLM, it utilizes a combination of high and low melting point powders, which results in a lower energy beam required for particle fusion [71]. The SLS printer involves an enclosed chamber containing three main components, i.e., the laser source, powder spreading platform and powder solidifying bed [72]. A moving roller transfers powder from a delivery platform to a solidifying platform, where it is selectively heated by a laser system that draws shapes layer-by-layer, causing fusion between the particles. The roller (or a blade) redistributes the powder after each layer. The process of the powder distribution and laser processing is repeated until the completion of the three-dimensional object [73,74,75]. Following the sintering process, an object is cleansed from unexploited remains by compressed air and may necessitate post-processing [76]. The SLS-processable materials in biomedical engineering include polymers, ceramics, biomaterials, and composites, e.g., ABS, PEEK, PMMA, hydroxyapatite, cellulose, glass-ceramics, calcium phosphate, and poly-L-lactic acid [77]. It can also use pure titanium or alloy powder, such as Ti-6Al-4V [78]. The quality of the SLS products contributes to their widespread commercial distribution [79], provided by its structural freedom of residual stress and internal defects. SLS is used in the manufacturing of medical devices and pharmaceutics, e.g., disc matrices for drug delivery devices [80,81]. SLS-fabricated surgical instrumentation is attracting growing attention in the orthopedics industry [82]. SLS can be used to produce various endoprostheses [83,84]. In the context of cranioplasty, Bertani et al. described the technique of printing cranial prosthesis’ molds made from polyamide (PA12) with a subsequent PMMA-made final implant preparation. According to this study, the total time required for implant preparation has not exceeded 42 h and has resulted in a 15-fold decrease in the total costs in comparison to the other methods [85]. Titanium implants, manufactured by selective laser sintering, were used in patients with Chiari malformation Type 1 after a suboccipital craniectomy [86].

## 4. Cranioplasty

Neurosurgical procedures occasionally lead to the formation of a bone defect within the cranial convexity. Craniectomies are predominantly performed to reduce intracranial pressure after trauma or as a part of radical tumor resection [87,88], and are customarily followed by cranioplasty [89,90]. A reconstruction of the cranial vault aims to re-establish the initial cerebrospinal fluid dynamics, restore esthesis as well as provide appropriate brain tissue protection, which is essential for further rehabilitation processes [91,92,93,94].

The restoration of the head’s natural symmetry improves a patient’s mental state and positively affects further psychosocial development [95]. Cranioplasty is also the method of choice for the syndrome of the trephined, characterized by various neurological and behavioral disorders [96], concerning up to 24% of those patients suffering from cranial defects [97]. 

The available novel literature does not deliver a universal consensus regarding optimal cranioplasty timing [98]. Normally, it varies on clinical circumstances and substantially depends on the initial surgery’s indication. Routinely, the procedure is scheduled at a 3-month interval, especially in patients with a post-decompressive neuroinfection or in the case of treatment-resistant intracranial hypertension. According to some sources, early cranioplasty improves the dynamics of cerebrospinal fluid circulation [99]. Other authors highlight no difference in infection rates but underline better outcomes in cognitive function as well as wound healing [100,101,102]. Nevertheless, recent studies indicate that there are no statistically significant differences in the frequency of hydrocephalus morbidity between an early and delayed cranioplasty following a decompression craniectomy [99]. 

The cranioplasty procedure has since evolved from when Dutch surgeon van Meekeren described the first-ever successful bone transplant that was performed by using canine bone as an implant in 1668 [103]. Nowadays, a vast range of methods and materials significantly enlarge surgeons’ artillery. To cover the cranial defects, there is a choice of allografts, autografts, xenografts, or other bone substitutes [104,105,106,107]. Cranioplasty with the use of bone, derived from decompressive surgery from the same patient via allograft, is often associated with bone resorption, auto-immunologic reactions and a high infection risk [108,109], and is often not feasible due to skull fractures.

There is a wide variety of substitute materials available on the market. One of them is methyl methacrylate, which is a polymerized ester of acrylic acid [110] (PMMA). It provides strength, comparable to natural bone tissue, and allows the prosthesis to be accurately adjusted to the shape of the defect. Its biggest and most significant disadvantage, from a clinical point of view, is its greater susceptibility to infections [103]. Another example of useful material is calcium phosphate bone cement [111,112]. Calcium phosphate bone cement is readily used for minor craniofacial defects and, in the case of cranioplasty, is often implemented in the treatment of skull defects in pediatric patients because, unlike methyl methacrylate, it does not prevent further expansion of the growing skull. In addition, some materials from this group, including those containing hydroxyapatite, possess osteoconductive properties [113,114]. Another material frequently used in cranioplasty is titanium, which can be used alone or together with methyl methacrylate. Titanium is noninflammatory, noncorrosive and nonferromagnetic and provides satisfactory cosmetic results [94,115]. 

Various techniques exist for bone flap fixation procedures. Ideally, it should ensure proper stabilization, a good cosmetic effect, minimize artifacts in neuroradiological imaging and be inexpensive [116]. The most described methods in the literature utilize sutures, wires, or mini plates, each of which has advantages and weaknesses. Among the above-mentioned, titanium mini plates seem to be the most beneficial due to their high biocompatibility and favorable osteointegrative properties [103,117,118] (Table 2). 

A retrospective review based on 329 medical cases showed that the most common complications after a cranioplasty procedure are infections (39%), followed by incorrectly fitting implants (30%). Furthermore, there is a risk of cerebrospinal fluid leakage and cerebral edema (5%) [119]. However, adverse effects may occur regardless of the type of prosthesis material.

The risk of infection depends on the material used during the procedure, the location of a previous craniectomy and appropriate wound care following surgery. Overall, according to a systematic review designed by van de Vijfeijken, autografts carry higher complication and failure risks in comparison to allografts [106]. 

## 5. 3D Cranioplasty

3D printing, which is gaining increasing popularity in various fields of science, has also found an application in medicine. It is used by various specialists, including neurosurgeons. Thanks to this technology, it has become possible to print precisely fitted, low-cost bone prostheses for patients requiring postoperative cranioplasty [30,31,123]. It is also used in surgeries for pre-planning incision lines, which allows the operators to perform a precise opening of the skull, reducing the risk of complications and improving the cosmetic effect [22]. In justified cases, print castings can be used to reconstruct a cranial base, for instance, in Arnold–Chiari syndrome [29]. Modern techniques for the repair of sphenoid dysplasia are also based on the pre-operative 3D-printing models of the skull base [32,124]. The use of 3D printing also contributes to the preparation of autologous transplants for craniofacial reconstruction surgeries in patients after severe burns [125]. Prostheses made with the use of 3D technology have high aesthetic value, which has become more important for patients nowadays [126]. In combination with the CAD software, it opens the way for the reconstruction of the continuity of the bone involving even complicated anatomical structures, e.g., the orbit [127,128]. The method employed to design a 3D image and replicate it to a 3D virtual model is called reverse engineering [129]. In addition to its universal availability, 3D printing allows for the preparation of personalized, perfectly-sized bone prostheses, which significantly reduces the time of surgery compared to the procedures that utilize standard implants. As an example, an intraoperative placement of the tegmen plate in temporal bone defects can be shortened from 60 to less than 1 min [130].

The improvement in 3D-printing techniques, the introduction of newer advanced materials and the easy availability of affordable 3D printers have made the creation of 3D-bone prostheses easy and generally available for hospitals. The relatively uncomplicated production makes it possible to create personalized bone prostheses, even on the premises of a medical facility. Creating an appropriate design and printing a prosthesis saves time, both at the preoperative stage and during the procedure of applying it to the patient, thanks to the precise adjustment to the bone defect. The employment of 3D printing for cranial defect implant fabrication has some limitations. The most important factor is the choice of appropriate material that gives the best end result in terms of its expense, regulations, durability, function, and safety [131]. Selecting appropriate material reduces the time of patient care and enables patients’ faster recovery [132,133,134]. An ideal implant is inert, biocompatible, eligible for sterilization, strong and possesses long-term stability, durability, and intraoperative workability [135].

In this article, we aimed to address these concerns and analyzed the available materials that are commonly selected for the prosthetic 3D-printing market: PLA, PET-G, ABS, PEEK, and PMMA.

### 5.1. PLA

Polylactic acid—PLA—is a versatile biopolymer classified as an aliphatic polyester. PLA is an FDA-approved, non-toxic, biodegradable material that is widely used in a plethora of applications, including in the biomedical and automotive engineering industries [136,137,138,139]. In healthcare, it finds employment, for example, in regenerative medicine, cancer therapy, tissue engineering, or cardiovascular implants, naming a few [140]. Currently, PLA is gaining rising attention because of its excellent performance as a three-dimensional printing material, especially with the employment of FMD technology [141], which is attributable to its mechanical properties. Polylactic acid is characterized by a low melting point (about 220 °C) as well as a low level of distortions during the printing process [142]. In tests, PLA shows two times greater resistance to bending and pressure than ABS [143]. Nonetheless, its poor toughness with a 10% elongation at break prevents its use when high-stress resilience is to be expected [144]. In the medical field, PLA shows potential in radiotherapy, as it appears to be undamaged by radiation exposure [145]. PLA blended with hydroxyapatite may potentially be used to repair bone defects [23,146,147]; however, there is little research and information on the ability of HA/PLA to stimulate bone formation in vivo [148]. PLA might also be used for anatomical model preparation and preoperative planning [149,150]. In addition, polylactic acid, along with PLGA—poly(lactic-co-glycolic acid)—represents the polymers most abundantly fabricated to create biodegradable scaffolds for the regeneration of cranial bone [51,151]. The novel literature delivers several examples of PLA employment in cranioplasty surgeries. De la Pena et al. used a 3D-printed-PLA calvarial defect model to create a plaster mold over it. The mold was then used as a form for the ultimate PMMA implant [121]. Other authors decided to print a 3D-PLA mold directly, and again, PMMA was used as a cranial prosthesis [152,153]. Similarly, Lannon et al. used desktop 3D printers to produce a PLA-made patient-specific mold for a PMMA-made final cranial implant and reported a significant reduction in the total procedure’s expenditure [154]. None of the mentioned authors experienced any significant complications that would stand out in comparison to the standard cranioplasty methods. The overall complication rate is difficult to assess due to the small number of patients involved. 

**Chart 5:**
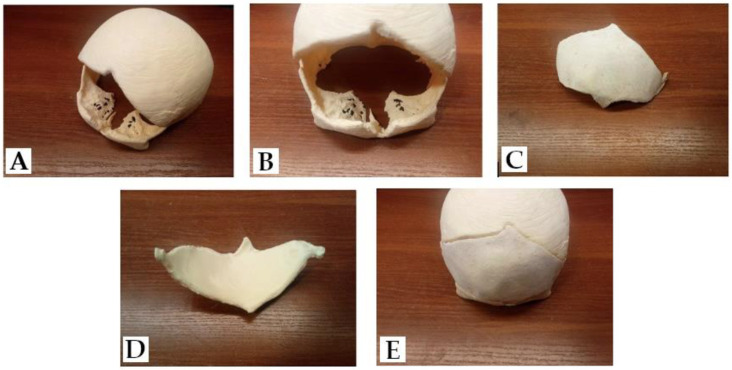
(**A**,**B**) CT-derived FDM-printed PLA model of a complicated skull defect post-craniectomy. (**C**,**D**) Implant ready for cranioplasty; (**E**) model simulating cranioplasty’s procedure outcome.

In Macedonia, Apriawan et al. implemented PLA as a material for the cranioplasty of two patients following decompressive craniectomies. In both cases, no postoperative complications have been recorded [155]. A simulation of cranial reconstruction with a use of PLA is presented in Chart 5 below.

### 5.2. PET-G

PET-G, an amorphous thermoplastic polyester resin belonging to the PET family and one that is subjected to secondary glycol modifications, is another option. It is characterized by its good impact strength, transparency, and chemical resistance [23]. It is also approved by the FDA as a water and food container [156]. The glass transition temperature of PET-G is around 80 °C, making it suitable for thermal forming [147], but unfortunately, it is not autoclave-resistant. It is considered an improvement over ABS due to more flexibility and biocompatibility [157]. In medicine, PET-G is used in ligament [158], joint [159] or vascular [160] prostheses. Additionally, PET-G prints can be potentially used to produce artificial breasts for mammography imaging techniques [161]. Katsching et al. successfully printed well-fitting, mechanically stable, and cleanly printed implants for maxillofacial defects ex vivo [162]. Although it has been used as a prosthetics material in various branches of medicine or for bone tissue engineering [163], we found no studies where PET-G was used as a direct material for cranioplasty in vivo. Nevertheless, there are multiple studies where PET-G was utilized to create a mold for the PMMA cranial implants. Kim et al. [164], Parthasarathyet [165] as well as Surme [166] used various techniques of mold preparation, whereas they all reported the avoidance of local and systemic PMMA-associated toxic adverse reactions as well as brain or dural tissue destruction. In each study, the operation time was significantly decreased with the preservation of high aesthetic value.

### 5.3. ABS

Products made from ABS, i.e., acrylonitrile butadiene styrene terpolymer, also show satisfactory durability. They are stiff but still more malleable than PLA [167]. In comparison to PLA, ABS is more difficult to print due to its greater expansion at higher temperatures [168]. In addition, it produces toxic fumes during the printing process [169]. The printing temperature for ABS and PET-G is similar, around 240 °C [170]. ABS implants are inert, biocompatible and permit tissue integration [171]. Ziąbka et al. explored ABS’s properties for the purpose of middle ear implant surgeries, where ABS proved to have great biocompatibility as well as low cytotoxicity [172]. Sarah E. Mowry used ABS to replicate human temporal bone for surgery simulation purposes. According to the evaluating specialists, the ABS-3D model proved to be accurate and may serve as a valuable alternative for cadaveric training [173]. In 2020, the FDA approved ABS as a low-risk polymer when in contact with intact skin. Kinsman et al. utilized ABS to prepare a patient-specific 3D model, which was used to customize the titanium mesh preoperatively, excluding the need for intraoperative molding [174]. The method proved to be resource and time-saving in comparison to the use of prefabricated personalized PEEK implants. ABS plastic, due to its mechanical properties as well as the printing method, is not an exquisite candidate to be used as an implant [175], although it might be successfully used as a material for PMMA molding. Yerragunta et al. prepared a 3D-printed ABS template for molding PMMA in 10 patients with post-craniotomy defects, reaching excellent calvarial contour restorations as well as patient satisfaction [176]. No long-term complications or infections have been noted on a follow-up. The same method was chosen by Unterhofer et al. and was conducted on 46 patients with 41 months of mean follow-up. Among these, 7 required reoperations, but overall, 42 achieved excellent cosmetic results [120]. These lead to a conclusion that the fabricated in-house PMMA implants, by direct molding with the use of the ABS model, are easy to produce, provide satisfying aesthetic results and are inexpensive in comparison to the industrially manufactured personalized cranial implants. 

### 5.4. PEEK

PEEK (polyether ether ketone) is a poly-aromatic, partly crystalline thermoplastic polymer increasingly used in orthopedics and dentistry [177,178,179,180]. The melting temperature of PEEK is around 343 °C [181]. The tensile strength is comparable to enamel, and its wear resistance is similar to metal alloys [182,183]. PEEK is inert and noncytotoxic; however, it is hydrophobic, making it susceptible to the adhesion of bacterial cells [184]. Platelet adhesion to PEEK is much lower than to polished medical steel and is similar to titanium alloys [185]. Because of the lack of osteogenic properties, it is more prone to infection and displacement [103]. Unfortunately, it is also considered the most expensive among other types, which limits its commercial dissemination [60,106]. It has great strength and a low Young’s modulus, comparable to human bone [104,186], which allows for an equal dispersion of induced stress and limits osteolysis [187]. It is also radiolucent and CT and MRI compatible, and due to its properties, it can be sterilized and re-sterilized in an autoclave without deformations [188]. The use of PEEK in combination with CAD technology allows us to obtain very satisfactory aesthetic effects in the production of bone implants, e.g., those used in maxillofacial surgery [189]. Nevertheless, the novel literature presents scarce examples of PEEK applications as a material for cranioplasty, regardless of its undeniable potential. According to Thien et al., cranioplasties with PEEK exhibit a lower incidence of revision surgeries (12.5%) than those with the use of titanium implants (25%) [190]. Meta-analyses performed by Punchak et al. and van de Vijfeijken proved that surgeries performed with the use of PEEK carry lower risks of infection than PMMA and autologous bone cranioplasties [106,191]. In a study performed by Mian et al., 3D-printed PEEK implants delivered great precision, minimal deviation (0.5919 mm), with maximal von Mises stress (8.15 MPa), von Mises strain (0.002) and minimal deformation (0.18 mm), which underlines the implant’s extraordinary load-bearing capability [192]. Similar observations have been made in a preclinical study designed by Sharma et al. [193]. De Barros described a one-step customized PEEK cranioplasty, reaching a favorable outcome without any major complications during a 3-month follow-up [194]. Mohsen et al. evaluated 20 patients who underwent a cranioplasty with prefabricated-3D-printed PEEK implants in the years 2017–2018. In this study, excellent cosmetic results were achieved by all participants. Apart from one superficial wound infection and one clinically irrelevant extradural hematoma, no major complications have been encountered [195]. 

The data from the current literature are encouraging, but the routine use of 3D-printed PEEK cranial implants requires further studies and is limited due to a lack of testing and validation. This might be accelerated by pending studies, which aim to establish the safety and efficacy of the house-manufactured PEEK implants used in cranioplasty in comparison to conventional methods [196].

### 5.5. PMMA

PMMA—(polymethylmethacrylate)—is a popular resin that is strong, light, stable and nonferromagnetic [197,198]. Among the alloplastic materials, PMMA is the most widely used by prosthesis manufacturers because it is less expensive than PEEK and titanium, which brings additional importance to its value in countries where funds reserved for the implant must be covered by the patient’s own resources [121]. Adding PMMA powder to other additive manufacturing processes can increase the porosity of parts and thus increase bone ingrowth [199]. PMMA, due to its high biocompatibility, is a useful material in dentistry and orthopedics [200,201,202,203]. Prostheses made with this technique adequately protect the tissues and present a mechanical strength analogous to natural human bone [122]. In addition, PMMA-based bone cement can be used to stiffen endoprostheses with the patient’s bone [204,205]. 

The PMMA castings built on printed forms can be further refined, e.g., by drilling holes, thus enabling a better flow, adhesion to soft tissues and providing greater stability [31]. 

Apart from their favorable mechanical properties, the advantages of PMMA implants also include their permeability for imaging studies [206]. Unfortunately, they can sometimes cause an immunologic reaction in the surrounding tissues, leading to subcutaneous seroma [207]. Moreover, the placement of polymers and their slow release of toxic monomers seldom initiates hypersensitivity [123,208]. 

The review of 41 articles regarding cranioplasty materials presents that there is no significant difference in the average infection rate for autologous bone (10.5; relative risk = 1.00) and hand-formed PMMA implant (10.98; relative risk = 1.05). In comparison, a prefabricated PMMA’s relative infection risk reaches about 0.66 [209,210,211]. Apart from the possible complications after implanting the 3D-printed bone prosthesis, it is difficult to predict its mechanical properties. The resulting strength is highly dependent on the method of printing and on a variety of parameters, which can be selected in accordance with a contractor’s experience. Material shrinkage greatly affects the accuracy of a material, as well as the errors induced by the CAD/CAM software and post-processing. The size of a printer restricts the size of the implant; however, in the case of cranial bones is not a remarkable limitation [4]. 

The available literature delivers numerous examples of PMMA applications in 3D cranioplasty. The safety and efficacy of long-term use of PMMA prostheses covering large cranial defects were assessed and approved after a long-term follow-up of randomized, blinded, multi-centered trials conducted by Jaberi et al. [212,213,214]. Morales-Gomez examined 22 patients who underwent cranioplasty that utilized PMMA with the use of a desktop 3D printer. Apart from a great cosmetic result achieved, no infection, implant rejection or wound dehiscence were reported in the 6-month follow-up [215] Yerragunta et al. used the same method in India and achieved comparable results, determining this technique as the most economically effective among all [176].

Dabadi et al., in a study conducted in Nepal, implemented 3D-printed PMMA cranial implants made on a silicon mold in three cases in patients after craniectomies in search of improved efficacy, expense- and time-wise. This has been achieved with no major complications reported with the acquisition of a great aesthetic and economic outcome [216]. 

## 6. Sterilization

FDM-printed parts can be sterilized in an autoclave, although it links to a 0.1–3% dimension shrinkage, which must be considered during manufacturing [217,218]. A similar phenomenon occurs in SLA-printed parts. The autoclaved prints present unwanted bends, causing displacement of the SLA-printed surgical guides, and contain numerous cracks [219]. 

Hydrostatic high-pressure post-processing may be a promising alternative to traditional sterilization techniques. Overall, it preserves better tensile properties than autoclave. Unfortunately, the DLP-processed samples are still damaged in the sterilization process, and thus, the mechanical properties are drastically decreased. Microbiological screenings in HHP adhere to the Good Laboratory Practices; however, further evaluation should be executed [220]. Titanium alloy implants fabricated with the SLM technique can be fully sterilized, both by dry heat and steam. However, there is no available ISO protocol for porous implant sterilization [221]. 

PLA, in consequence of a relatively low melting point (180–220 °C) as well as glass transition temperature (57 °C), is not applicable for autoclave sterilization. Nevertheless, PLA implants can undergo a sterilization process with the use of a low-temperature hydrogen peroxide gas plasma system (HPGP) and new scCO_2_ (supercritical carbon dioxide) technique [222]. Aguado-Maestro suggests EtOH sterilization for in-hospital 3D-printed PLA materials, whereas, for gas plasma, an infill application of 100% should be applied [221]. While PET-G is not eligible for autoclave sterilization, ultraviolet radiation sterilization, and ethylene oxide sterilization provide adequate alternatives [162]. Dautzenberg reported the instability of ABS following steam sterilization [217]. According to Toro et al., vaporized hydrogen peroxide sterilization provides dimensional stability for sterilized ABS-3D printed elements [223]. The same was reported by other authors [224,225]. PEEK implants are known to retain their mechanical properties after multiple steam sterilization cycles [226,227]. Münker et al. inspected the micromechanical properties of PMMA after various sterilization systems and reported significant changes in the material’s mechanical behavior after autoclave sterilization. A favorable outcome was achieved with the use of ethylene oxide, hydrogen peroxide gas plasma and γ-irradiation sterilization [228].

## 7. Costs and Legal Regulations

The 3D-printing market share grew to USD 5.165 billion in 2015 and is predicted to reach USD 30 billion in 2022 [229].

Pricing of a 3D printer ranges from a few hundred for low-end devices to hundreds of thousands (USD) for industrial printers. An affordable, ready-to-use Ultimaker, which costs around USD 2750–3000, is fully capable of printing a human skull model. The 3D-printing market offers an abundance of materials, with the simplest Poly(lactic acid) filament for as low as USD 20 per 1 kg, with an average cost for the 3D-printed craniofacial models ranging from USD 20 to 100. In a study conducted by Apriawan et al., the PLA cost for a 3D-cranioplasty model reached around USD 50–150 [155]. A PLA-made-cranial model for surgical consulting purposes costs around USD 5.20 [230]. The cost of a 3D printer and ABS (acrylonitrile butadiene styrene) oscillates around USD 1350 and USD 25/kg, with one kg of ABS being able to generate around 7–10 3D models [174]. A higher price is usually associated with the more specialized printing processes. In a study by Unterhofer et al., conducted on 46 patients, the expenditure of PMMA-implant production oscillated around EUR 81 [120]. For comparison, the manufactured personalized PEEK implants cost around USD 10,450, titanium implants cost USD 10,265 and HA is USD 7849–8960 [60,155] (Table 3).

Ballard et al. calculated the potential economic profits related to the additive manufacturing of cranial prostheses. Assuming the average operating theater cost exceeds 62 USD/min, the possible net savings were estimated at USD 19,284–USD 129,586 for a low (5%) and USD 77,536–USD 518,358 for a high (20%) utilization rate [240]. 

In some countries, i.e., Mexico, cranioplasty procedures are not refunded by the national health service, and the cost of the operation, as well as the implant, needs to be covered by the individual. With the cost of widely used personalized titanium implants that reach around USD 5000 and those made of PEEK of around USD 7000, it is undemanding to assume that these prices reach financial levels that are not achievable for medium- to low-income patients. For comparison, a personalized prosthesis, created with 3D-printing technology and comprised of PMMA, wavers around USD 600, including the 3D design, prototype, and the prosthesis itself, which diminishes the overall expense by over tenfold [121,241]. Around 85 3D-printed devices were approved by the FDA for medical purposes through 510(K) processes [229]. The 510(K) is a premarket notification—another pathway to apply for an NDA (new drug application). Other pathways include premarket approval (PMA) and a biologics license application (BLA) [239].

## 8. Conclusions

Cranioplasty is a common procedure performed by neurosurgeons all over the world. In the cases of a complicated, uneven, and large deficit, where a random-sized prosthesis is not sufficient, personalization of the material is inevitable. Unfortunately, this comes with a vast increase in price, even 5- to 10-fold. Therefore, it is crucial to obtain a method that allows us to manufacture complicated, personalized cranial prostheses that are designed for departments located in developing countries and patients with a low socioeconomic status. This is achievable due to the development and spread of 3D-printing technology.

**Chart 6:**
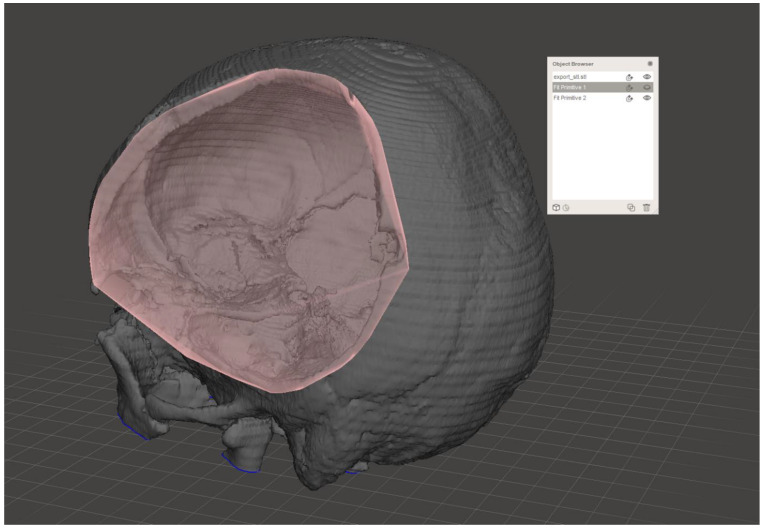
Automatically generated part is user-checked on the fly, adjusted using tools provided with Autodesk^®^ Meshmixer and exported to printable file.

There are numerous factors that influence the cost of the prosthesis, i.e., size, type of material and manufacturing technology. A low-cost prosthesis can be successfully made using home and even DIY printers as well as typically industrial and professional devices, such as Stratasys^®^. The market offers a wide range of CAD software which enables the personalization of an implant (Chart 6). It is even possible to design and prepare the model by using free software solutions. Apart from its employment in cranioplasty, 3D-assisted preoperative planning has become particularly popular, allowing surgeons to better understand the site of the planned reconstruction. Three-dimensional printing often allows us to obtain satisfactory cosmetic effects, restoring the correct skull contour and thus increasing the patient’s quality of life. In fact, there is no shortage of companies dealing with the more expensive technologies, such as the production of titanium prostheses; however, there is a niche for start-ups with low-share capital who are aiming to provide services for patients living in poorer regions. A plethora of various technologies of additive manufacturing exist, so it is currently difficult to decide which one of them will prove to be the best in the future, as in many cases, the research is still pending. As 3D-printed PMMA could be considered too expensive, more attention should be pointed to using prints from the PLA in the PMMA prosthesis creation process, especially due to the material’s satisfactory strength and low manufacturing price. This method has been attempted in several cited publications, achieving satisfactory results in terms of cost, esthetic value, as well as outcome. 

In conclusion, 3D-printed cranioplasty might be an interesting, efficient, and inexpensive alternative to the methods that are currently routinely used. 

## Figures and Tables

**Table 1:** Summary of properties of the 3D-printing technologies.

Technology	Materials Used	Printing Technique	Application in Medicine
Selective Laser Melting (SLM)	metal alloys (e.g., titanium)	processing with a laser beam	screws hip prostheses [18]
Fused Filament Fabrication (FFF, FDM)	PLA, ABS, PET-G, PP, PMMA, PEEK, PVDF [19]	forming layers by depositing material through a heated, moving nozzle	models for preoperative planning [20,21,22,23] bone scaffolds and implants [24] cartilage repair [25,26] “drug delivery” [27,28] cranioplasty [29,30,31,32]
Stereolithography (SLA)	photosensitive polymers	focusing a UV laser onto a vat of polymer resin	endocardial implants [33] models reconstructing the intestinal epithelial architecture [34] treatment of focal cartilage lesions [35]
Digital Light Processing (DLP)	photosensitive polymers	like SLA, but instead of the laser beam, the entire layer is cured at the same time	exoprotheses used in the rehabilitation [36] dentures [37]
Selective Laser Sintering (SLS)	PA11 (nylon), PA12, PS, EOS TPE, PEEK	sintering with a laser beam	dental industry [38]

**Table 2:** Comparison of bone-substitute materials used for cranioplasty.

Properties	PMMA	Hydroxyapatite	Titanium
Physical	non-conductive [103,120,121] radiolucent [103,120,121] good mechanical resistant and compression properties [103]	non-conductive [113] radiolucent [113] low tensile strength and mechanical resistance [103,113]	heat and cold conductive [103,113] non-radiolucent [113] excellent mechanical strength [113]
Biocompatibility	Good [120]	High [103]	High [103]
Risk of fracture, fragmentation	High in larger defects [103]	High [103]	Low [113]
Osteointegration	Poor [106,113]	Excellent [103,113]	Poor [113]
Intraoperative modifying	Yes	Yes	No
Accommodation with skull growth (pediatric use)	No	Yes	No
Infection rate	5.8–12.7% [103,113,122]	0.6–2.1% [111,112,113]	2.6–5.4% [103,112,113]
Mean implant cost	$80 (for intra-op. molded version) $650–$800 (for CAD/CAM version) [120,122]	$7900 [120]	$25,100

**Table 3:** Characteristics of 3D-printed materials.

	Injection-Molded PMMA	Injection-Molded PEEK	3D-Printed PET-G	3D-Printed ABS	3D-Printed PLA
Used for 3D cranioplasty as	Implant	Implant	Mold	Mold	Mold
Tensile strength [MPa]	48–76 [231]	80 [231]	50 [232]	37 [233]	47–52 [234]
Elastic modulus [GPa]	3–5 [231]	3–4 [231]	1.1–1.3 [232]	1.8 [143]	3.4–5.7 [234]
Biocompatibility	Good [177,235]	High [231]	Yes, in specialized filaments [163,236]	Yes, in specialized filaments [172,236]	High (in pure materials) [236]
Osteointegration	Poor [115,123]	Poor [231]	Under research [163]	Under research [172]	With coating [237]
Complication rate [%]	7.4 [231]	9.1 [231]	N/A	N/A	N/A
Cost of material [USD per kg]	30	275–400 [238]	40	25 [175]	20
Total cost of 3D-printed implant manufacturing [USD]	600 [152,239]	10,450 [156]	N/A	N/A	N/A

Abbreviations: PMMA = polymethylmethacrylate; PEEK = polyether ether ketone; PET-G = gylocol-modified polyethylene terephthalate; ABS = acrylonitrile butadiene styrene; PLA = polylactic acid; N/A = non-applicable.

## Abbreviations

ABS	Acrylonitrile butadiene styrene	CAD/CAM/CAE	Computer-aided design/Computer-aided Manufacturing/Computer-aided Engineering
DICOM	Digital Imaging and Communications in Medicine	DLP	Digital Light Processing
FDM^®^	Fused deposition modelling	FFF	Fused filament fabrication
HA	Hydroxyapatite	PEEK	Poly(ether ether ketone)
PET-G	Poly(ethylene terephthalate glycol)	PLA	Poly(lactic acid)
PMMA	Poly(methylmethacrylate)	PP	Polypropylene
PVDF	Poly(vinylidene fluoride)	SLA	Stereolithography
SLM	Selective laser melting	SLS	Selective laser sintering
STL	stereolithography CAD software file format		
